# Screening for Dementia in Older Adults: Comparison of Mini-Mental State Examination, Mini-Cog, Clock Drawing Test and AD8

**DOI:** 10.1371/journal.pone.0168949

**Published:** 2016-12-22

**Authors:** Li Yang, Jing Yan, Xiaoqing Jin, Yu Jin, Wei Yu, Shanhu Xu, Haibin Wu

**Affiliations:** 1 Zhejiang Provincial Center for Cardiovascular Disease Control and Prevention, Zhejiang Hospital, Hangzhou, China; 2 Zhejiang Hospital, Hangzhou, China; 3 Zhejiang Provincial Center for Disease Control and Prevention, Hangzhou, China; Istituto Di Ricerche Farmacologiche Mario Negri, ITALY

## Abstract

This study was conducted to estimate screening performance of dementia screening tools including Mini-Mental State Examination (MMSE), Mini-Cog, Clock Drawing Test (CDT) and Ascertain Dementia 8 questionnaire (AD8) for older adults. 2015 participants aged 65 years or more in eastern China were enrolled. 4 screening tests were administered and scored by specifically trained psychiatrists. We used data from two-by-two tables to calculate the sensitivity, specificity, and positive and negative predictive values (PPV/NPV). Our study showed that dementia was highly prevalent among elderly in Zhejiang province. The Mini-Cog, with excellent screening characteristics and spending less time, could be considered to be used as a screening tool among communities to help to diagnose dementia early.

## Introduction

Dementia is common among older adults and is related to reduced quality of life and life expectancy [1,2]. It is one of the major economic burdens for public health systems. China is facing substantial challenges in ageing population, of which increasing numbers will have some degree of dementia [1,3,4].

Despite being incurable, there are several benefits of dementia screening earlier. It allows patients and their caregivers to make decisions regarding future planning [5,6]. And pharmacological treatments in time such as cholinesterase inhibitors or memantine may help slow the progression of dementia [7–9].

Dementia and mild cognitive impairment (MCI) are under-recognized in community settings [10]. This may be due in part to the lack of brief and suitable dementia screening tools available to general practitioners, although a growing consensus recommends routinely screening patients for cognitive impairment when they are over a certain age (e.g., 75 years) or when cognitive decline may be beneficial[11,12].

There are several dementia screening tools that would screening dementia early in the disease course, such as Mini-Mental State Examination (MMSE) [13], Mini-Cog [14], Clock Drawing Test (CDT) [15]and the Ascertain Dementia 8 questionnaire (AD8) [16].MMSE, the most commonly used instrument, shows education and language/cultural bias[17] and is impractical[18] because it takes at least of 10 minutes to complete[19]. Simple and effective instruments with administration times of five minutes or less seem most suitable for dementia screening [20].An appropriate screening tool has not only to be brief, unswayable, sensitive, but also has a high specificity [21].

The aim of this study is to estimate and compare screening performance of four existing dementia screening toolsincluding MMSE, Mini-Cog, CDT and AD8 based on cross-sectional dataset.

## Methods

### Study design and study population

**Fig 1:**
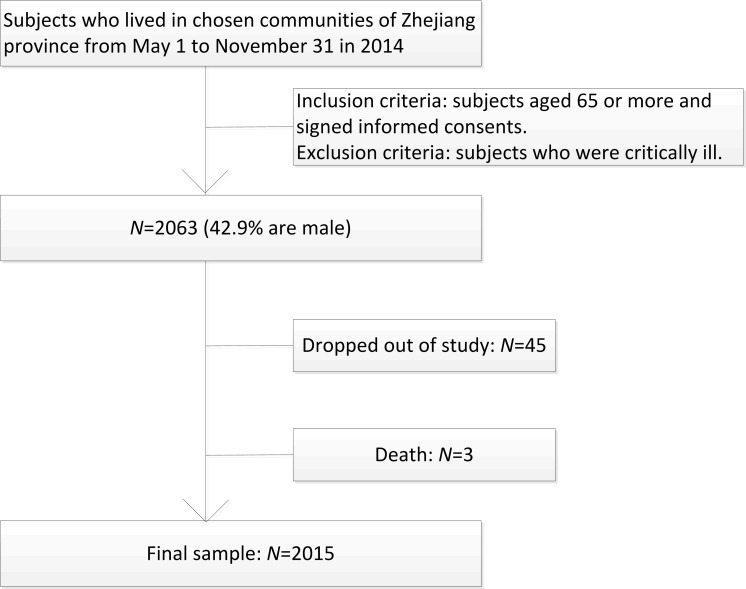
Schematic diagram of study participant selection.

This study was a cross-sectional survey administrated in 4 communities across 12 counties in Zhejiang province using the method of multi stage stratified random cluster sampling. First, 12 administrative districts were divided into 4 type districts based on economic levels. From each of these 4groups, 1district was systematically selected. Then 1community was randomly chosen from each district. Subjects aged 65 or more living in the selected communities were invited to participate (Fig 1).

The inclusion criteria: (i) subjects aged 65 or more,(ii) could complete CDT; and (iii) subjects or his/her relatives were aware of the study and signed the informed consent. Subjects were excluded due to these conditions: (i) having the history of acute severe disease, such as acute myocardial infarction, acute cerebral infarction and other severe heart, brain, kidney and liver diseases,(ii)unwilling to participate; (iii)severe deafness, or aphasia to communicate.

From May to November of 2014, 2,063 subjects were recruited, 45 were excluded because of low compliance and3 were because of death. A total of 2,015 subjects were included in the final analysis (response rate was 97.7%) (Fig 1).

### Ethical consideration

The protocol of this study was approved by the Medical Ethics Committee of Zhejiang Hospital. The participants were informed about the objectives and methods of the study. They were informed that their participation was totally voluntary and that they could withdraw from the study at any time without citing any reason. Written and signed or thumb printed informed consent was obtained from those who agreed to participate, or from their guardians. The methods used in this research were carried out in accordance with the approved guidelines.

### Measure

The survey was conducted in residence or institution of participants. The demographic information was obtained from subjects and caregivers or relatives, including age, areas of residence, education and health behaviors such as history of smoking, alcohol, tea consumption, occupation, family income and illness history. Physical examinations performed by trained nurses including height and weight were also collected. Tests and scales including MMSE, Mini-Cog, CDT and AD8 were administered and scored by specifically trained psychiatrists[2].

According to previous studies, dementia diagnosis was defined using NIA-AA criteria (2011) [2]. MMSE scored below 20among the illiterate (<1 year education) or below 23 among participants with primary school education level (1–6 years) or below 27 among participants with middle school or higher education level (>7 years) were considered as dementia [2].For the Mini-Cog analysis, the optimal algorithm had the following three rules: (i) subjects recalling none of the words were classified as having dementia, (ii) those recalling all three words were classified as without dementia, and (iii) those with intermediate word recall (1–2) were classified based on the CDT(abnormal = having dementia, normal = without dementia) [14].For the CDT evaluation in the present work, the results were evaluated by scoring from 1 point (perfect) to6 points (not at all representation of a clock), a score less than 4was considered as dementia.TheAD8 had 8 yes/no questions and took 2 to 3 minutes to complete [22], and a score of AD8 above 1 was considered as dementia [14].

### Statistical analysis

Epidata 3.0 was used for data entry and validation and SAS9.3 for data management and analysis. Socio demographic characteristics of participants were summarized using frequencies (percentages) or means and standard deviations, and they were compared by the Student’s t-test and χ2 test, respectively.

Age-gender-standardized prevalence rate was calculated by a direct method with a standard population (the sixth population census in Zhejiang, 2010).

We used data from two-by-two tables to calculate the sensitivity, specificity, positive and negative predictive values(PPV/NPV) as well as measures of statistical uncertainty (e.g. 95%confidence intervals, CI).We used the statistical software package SAS version 9.3 to derive statistics including the area under the roc curve (AUC) from ROC graphs.

Significance level was set at *P*< 0.05 for all hypothesis tests.

## Results

### Baseline Characteristics

A total of 2,015 participants (42.2% men) with a mean (SD) age of 79.5 (7.6) years were enrolled in the study. Among the total subjects, 444 (22.0%) were diagnosed with some form of dementia, the prevalence of dementia standardized by age and sex with a standard population (the sixth population census in Zhejiang, 2010) was13.0%.

Among the participants, there were 1165 (57.82%) women, 214 (11.6%) illiterate, 234 (13.4%) and 858(46.9%) having the habit of smoking and drinking, respectively. Compared with female subjects, the male had higher education levels and more were smokers. Meanwhile, there were less men living alone, having the medical history of coronary heart disease and having the habit of drinking tea. There were no statistically difference between male and female among the age and body mass index. The demographic characteristics of participants in different clusters such as gender, age and education level were showed in Table 1.

**Table 1:** Baseline socioeconomic characteristics with and without dementia among elderly of southern China in 2014 (n = 2015).

Characteristics	Women			Men			All			
	Without dementia	With dementia	Total	Without dementia	With dementia	Total	Without dementia	With dementia	Total	*P*-value*
Total, n(%)	880 (75.54)	285 (24.46)	1165 (57.85)	690 (81.27)	159 (18.73)	849 (42.15)	1571 (77.87)	444 (22.03)	2015 (100)	<0.001
Age,65–69,n (%)	126 (14.32)	5 (1.75)	131 (11.24)	116 (16.81)	9 (5.66)	125 (14.72)	242 (15.40)	14 (3.15)	256 (12.70)	0.707
70–74,n (%)	164 (18.64)	17 (5.96)	181 (15.54)	120 (17.39)	8 (5.03)	128 (15.08)	284 (18.08)	25 (5.63)	309 (15.33)	0.003
75–79,n (%)	205 (23.30)	35 (12.28)	240 (20.60)	144 (20.87)	26 (16.35)	170 (20.02)	349 (22.22)	61 (13.74)	410 (20.35)	0.001
80–84,n (%)	217 (24.66)	68 (23.86)	285 (24.46)	160 (23.19)	42 (26.42)	202 (23.79)	378 (24.06)	110 (24.77)	488 (24.22)	<0.001
85–89,n (%)	119 (13.52)	80 (28.07)	199 (17.08)	103 (14.93)	43 (27.04)	148 (17.20)	222 (14.13)	123 (27.70)	345 (17.12)	0.004
90–94,n (%)	43 (4.89)	60 (21.05)	103 (8.84)	39 (5.65)	23 (14.47)	62 (7.30)	82 (5.22)	83 (18.69)	165 (8.19)	0.001
95 and above,n (%)	6 (0.68)	20 (7.02)	26 (2.23)	8 (1.16)	8 (5.03)	16 (1.88)	14 (0.89)	28 (6.31)	42 (2.08)	0.123
Mean±SD (years)	78.08±7.04	85.03±6.85	79.78±7.60	78.15±7.49	83.34±7.10	79.12±7.69	78.11±7.24	84.42±6.98	79.50±7.64	0.056
Education, n(%)										
Illiterate (<1 year)	99 (11.25)	76 (43.68)	175 (16.60)	25 (3.62)	14 (14.89)	39 (4.97)	124 (7.89)	90 (33.58)	214 (11.64)	<0.001
Primary school (1–6 years)	247 (28.07)	47 (27.01)	294 (27.89)	116 (16.81)	36 (38.30)	152 (19.39)	363 (23.11)	83 (30.97)	446 (24.25)	<0.001
Middle school (7–12 years)	399 (45.34)	43 (24.71)	442 (41.94)	350 (50.72)	28 (29.79)	378 (48.21)	750 (47.74)	71 (26.49)	821 (44.64)	0.025
High school (>13 years)	135 (15.34)	8 (4.60)	143 (13.57)	199 (28.84)	16 (17.02)	215 (27.42)	334 (21.26)	24 (8.96)	358 (19.47)	<0.001
State of marriage, n(%)										
Married	544 (61.82)	53 (31.74)	597 (57.02)	571 (82.99)	57 (61.29)	628 (80.41)	1116 (71.13)	110 (42.31)	1226 (67.03)	0.376
living alone	336 (38.18)	114 (68.26)	450 (42.98)	117 (17.01)	36 (38.71)	153 (19.59)	453 (28.87)	150 (57.69)	603 (32.97)	<0.001
Medical history, n (%)										
Dementia	24 (2.73)	9 (5.63)	33 (3.18)	25 (3.63)	1 (1.08)	26 (3.32)	49 (3.12)	10 (3.95)	59 (3.24)	0.894
Coronary heart disease	388 (44.14)	58 (35.15)	446 (42.72)	256 (37.10)	26 (27.37)	282 (35.92)	644 (41.02)	84 (32.31)	728 (39.78)	0.009
Hypertension	594 (67.50)	95 (57.58)	689 (65.93)	472 (68.51)	56 (58.95)	528 (67.35)	1066 (67.90)	151 (58.08)	1217 (66.50)	0.526
Diabetes	176 (20.00)	39 (23.49)	215 (20.55)	134 (19.42)	27 (28.13)	161 (20.48)	310 (19.73)	66 (25.19)	376 (20.51)	0.970
Stroke	89 (10.14)	21 (12.57)	110 (10.53)	71 (10.30)	31 (31.31)	102 (12.94)	160 (10.20)	52 (19.55)	212 (11.56)	0.109
Family income, n(%)										
High	4 (0.45)	0	4 (0.34)	14 (2.03)	3 (1.89)	17 (2.00)	18 (1.15)	3 (0.68)	21 (1.04)	0.005
Middle	545 (61.93)	57 (20.00)	602 (51.67)	588 (85.22)	46 (28.93)	634 (74.68)	1134 (72.18)	103 (23.20)	1237 (61.39)	0.363
Low	331 (37.61)	228 (80.00)	559 (47.98)	88 (12.75)	110 (69.18)	198 (23.32)	419 (26.67)	338 (76.13)	757 (37.57)	<0.001
Lifestyle habits, n(%)										
Cigarette smoking	19 (2.17)	6 (3.59)	25 (2.39)	188 (27.29)	29 (31.18)	217 (27.75)	207 (13.21)	35 (13.46)	242 (13.25)	<0.001
Alcohol drinking	356 (40.59)	136 (81.93)	492 (47.17)	293 (42.46)	72 (75.79)	365 (46.50)	650 (41.45)	208 (79.69)	858 (46.91)	0.255
Tea drinking	453 (51.83)	60 (39.22)	513 (49.95)	296 (42.90)	38 (40.43)	334 (42.60)	749 (47.86)	98 (39.68)	847 (46.74)	0.035
Body mass index, Mean±SD (kg/m2)	22.12±3.76	23.72±2.21	22.18±3.71	23.28±3.20	24.51±2.11	23.28±3.20	22.61±3.56	23.72±2.25	22.64±3.53	0.123

*P-value is the significance of t-test or chi-square between total women and total men.

### Estimation of screening tests

A large-scale community-based cross-sectional study was conducted to evaluate parameters for dementia screening tests including MMSE, Mini-Cog, CDT and AD8 with the gold standard of NIA-AA criteria(2011). Of the 2015 subjects in the dataset, 705, 620, 737 and 686 subjects were diagnosed as positive using the MMSE alone, Mini-Cog alone, AD8 alone and CDT alone respectively. Using all tools, 433 subjects were diagnosed with dementia, while 1582 subjects were diagnosed with no dementia. The sensitivity and specificity of screening tools were estimated by two-by-two tables. The results showed that MMSE had higher sensitivity, and Mini-Cog had higher specificity, compared with other screening tools. The AUC of the 4screening tools (MMSE, Mini-Cog, CDT and AD8) were 87.11%, 86.52%, 85.94 and 84.02%, respectively. More detail information about the 4screening tests could be seen in Table 2.

**Table 2:** Screening test characteristics of MMSE, Mini-Cog, CDT, and AD8.

Variables	MMSE	Mini-Cog	CDT	AD8
Sensitivity, % (95% CI)	92.79 (90.03–95.91)	87.61 (85.92–90.02)	90.09 (88.14–92.24)	89.64 (87.54–91.24)
Specificity, % (95% CI)	81.35 (79.03–83.98)	85.30 (83.07–87.92)	81.77 (78.03–83.83)	78.42 (76.54–80.84)
Youden Index, % (95% CI)	74.14 (71.98–76.42)	72.91 (70.92–75.05)	71.86 (69.49–73.42)	68.06 (66.93–70.43)
PPV, % (95% CI)	58.44 (57.29–61.04)	62.74 (59.98–65.12)	58.31 (56.93–60.73)	54.00 (51.94–56.32)
NPV, % (95% CI)	97.56 (95.09–99.04)	96.06 (94.23–98.43)	96.68 (94.53–98.84)	96.40 (94.92–99.05)
AUC, (95% CI)	87.11 (85.31–88.93)	86.52 (84.43–88.51)	85.94 (84.04–87.93)	84.02 (82.04–86.13)

Abbreviations: PPV-positive predictive value; NPV-negative predictive value; CI-95% confidence intervals.

### Estimation for the combinations among screening tests

Table 3 and Table 4 showed the sensitivities and specificities for each combination of the screening tests. We summarized the results when the tests were evaluated in serial combination (positive when all tests were positive and negative otherwise) and in parallel combination (positive when at least one of the tests was positive and negative otherwise)[23].The serial combination between MMSE and CDT had AUC of 87.72%, which were higher than the others, and the combination of the 4tests had the highest specificity of 94.07%, but with low sensitivity and AUC(Table 3). In the parallel model, the combination between Mini-Cog and CDT had a specificity of 79.16%, which was higher than others, and the combination of 4tests had the highest sensitivity of 99.55%, but with the lowest specificity and AUC (Table 4). Compared with the estimation of 1test alone, the result suggested an apparent improvement in specificity / sensitivity, but at a cost in sensitivity / specificity, when 2ormore tests are used jointly. The combined use of the tests did not significantly improve the probability of screening capacity. More detail information about the combinations among the 4screening tests could be seen in Table 3 and Table 4.

**Table 3:** Diagnostic test characteristics of the serial mode combinations among screening tools*.

Combination among tests	Sensitivity (%)	Specificity (%)	Youden Index (%)	PPV, % (95% CI)	NPV, % (95% CI)	AUC, (95% CI)
MMSE + Mini-Cog	83.11 (81.34–85.32)	91.22 (79.04–93.39)	74.33 (72.13–76.39)	72.78 (70.48–75.92)	95.03 (93.83–98.74)	87.23 (85.02–89.31)
MMSE + AD8	84.46 (82.34–86.93)	88.92 (86.43–91.03)	73.38 (71.83–75.58)	68.31 (76.03–72.47)	95.29 (93.36–98.17)	86.71 (84.52–88.83)
MMSE + CDT	84.91 (82.21–86.43)	90.44 (88.72–92.13)	75.35 (73.02–78.24)	71.54 (68.93–73.38)	95.49 (93.37–97.42)	87.72 (85.62–89.83)
Mini-Cog + AD8	81.53 (79.21–83.45)	91.92 (89.03–93.28)	73.45 (71.93–75.43)	74.03 (72.34–77.04)	94.63 (92.57–96.64)	86.73 (84.51–89.04)
Mini-Cog + CDT	85.59 (82.94–87.43)	87.89 (85.39–90.30)	73.48 (71.23–75.42)	66.67 (64.93–69.06)	95.56 (93.53–97.39)	86.74 (84.62–88.83)
AD8 + CDT	82.66 (80.39–84.43)	90.69 (88.03–92.26)	73.35 (71.28–75.04)	71.54 (68.74–74.93)	94.87 (92.51–97.03)	86.71 (84.53–88.92)
MMSE + Mini-Cog + AD8 + CDT	76.58 (74.32–78.93)	94.07 (92.32–96.23)	70.65 (68.04–73.08)	78.52 (76.86–81.28)	93.42 (91.32–96.07)	85.34 (82.91–87.80)

*Positive when all tests were positive and negative otherwise.

**Table 4:** Diagnostic test characteristics of the parallel mode combinations among screening tools^#^.

Combination among tests	Sensitivity (%)	Specificity (%)	Youden Index (%)	PPV, % (95% CI)	NPV, % (95% CI)	AUC, (95% CI)
MMSE + Mini-Cog	97.30 (95.13–99.24)	75.43 (73.60–78.26)	72.73 (70.03–74.43)	52.81 (50.83–54.37)	99.00 (96.83–100.0)	86.42 (84.70–88.02)
MMSE + AD8	97.97 (95.33–99.93)	70.85 (68.03–73.28)	68.82 (66.87–71.30)	48.71 (46.26–50.83)	99.20 (97.31–100.0)	84.43 (82.71–86.13)
MMSE + CDT	97.97 (95.37–99.71)	72.66 (70.05–74.32)	70.63 (68.52–72.34)	50.35 (48.63–52.32)	99.22 (97.41–100.0)	85.31 (83.63–87.04)
Mini-Cog + AD8	95.72 (93.47–97.63)	71.80 (68.93–73.35)	67.52 (65.41–69.04)	48.96 (46.81–51.15)	98.34 (96.63–99.99)	83.82 (81.91–85.62)
Mini-Cog + CDT	92.12 (90.26–94.31)	79.16 (77.42–82.14)	71.28 (69.43–73.73)	55.57 (53.22–57.32)	97.26 (95.74–99.99)	85.63 (83.72–87.53)
AD8 + CDT	97.07 (95.31–98.94)	69.47 (67.33–71.53)	66.54 (64.25–68.94)	47.36 (45.32–49.63)	98.82 (96.24–100.0)	83.31 (81.52–85.13)
MMSE + Mini-Cog + AD8 + CDT	99.55 (97.42–100.0)	63.16 (61.24–65.31)	62.71 (60.81–64.54)	43.33 (41.27–45.86)	99.80 (97.35–100.0)	81.42 (79.61–83.24)

^#^Positive when at least one of the tests were positive and negative otherwise.

## Discussion

The detection and early diagnosis of dementia are becoming increasingly important as the ageing of our population [11, 14]. Early detection of dementia in a preclinical phase is very important and a quick, cheap and practical screening test for dementia among healthy people is needed [24]. Using brief cognitive screening tools enabled us to detect dementia and mild cognitive impairment at the earliest stages [22, 25].

An appropriate screening tool had not only to be brief, sensitive, but it also had to show a high specificity. In daily clinical practice, a large portion of older adults sought help from a general or a memory clinic due to self-observed or by proxy observed memory complaints. A high specificity made practitioners differentiate dementia and “pseudo-dementia” easier.

This study estimated the diagnostic performance of MMSE, Mini-Cog, CDT and AD8 tools for dementia screening. To our knowledge, this study is the first to estimate the performance of the 4screening tests for dementia through community-based data in eastern China.

In our study, AUC of the serial combination of MMSE and CDT was higher than others (87.23%), but it may spend more time. AUC of the Mini-Cog was 86.52% with a high specificity of 85.30% and sensitivity of 87.61%. It was quick and easy to administer, which needed only 2–4 minutes to complete compared with more than 10 minutes of MMSE, while having psychometric properties similar to the MMSE. All of that made the Mini-Cog look like more suitable to be used as a screening test among apparently healthy elderly in communities.

This result suggested an apparent improvement in sensitivity/ specificity, but at a cost of specificity/ sensitivity. The combined use of the tests did not significantly improve the probability of identifying elderly with dementia, but spending more time to administrate.

The prevalence of Zhejiang province in our study was 13%, higher than the national survey conducted among Chinese people aged 65 and older (5.14%) [2, 26].In the investigation, we also found that few of the dementia patients were treated regularly with anti-dementia drugs, and none of them received cognitive rehabilitation therapies. More population based strategies to control and prevent dementia, including conducting community-based regularly screening dementia, carrying out primary prevention policies and public health services are needed to address this serious problem.

The present study had a number of strengths, including providing regional representative data at an elderly population level in Zhejiang province. Furthermore, we estimated the performance of 4dementia screening tools including MMSE, Mini-Cog, CDT and AD8together. Nevertheless, there were limitations that warrant consideration. Our study did not collect enough data to implement health economic evaluation for different screening tools of dementia. And this study was performed only in eastern China.

## Conclusions

In conclusion, we first tested the diagnostic performance of 4dementia screening tests including MMSE, Mini-Cog, CDT, AD8 tools and different combinations of the 4 tools for elderly through community-based data. Dementia was highly prevalent among elderly in eastern China. The Mini-Cog with excellent screening characteristics and spending less time, maybe should be considered to be used as a screening test to help to diagnose dementia early.

## Supporting Information

S1 Dataset The dataset of the manuscript. (XLS)
